# Radiofrequency ablation induces tumor cell dissemination in a mouse model of hepatocellular carcinoma

**DOI:** 10.1186/s41747-023-00382-5

**Published:** 2023-11-29

**Authors:** Bowen Zhuang, Xi Zhu, Jinhua Lin, Fuli Zhang, Bin Qiao, Jihui Kang, Xiaohua Xie, Xunbin Wei, Xiaoyan Xie

**Affiliations:** 1 Department of Medical Ultrasonics, The First Affiliated Hospital of Sun Yat-Sen University, Institute of Diagnostic and Interventional Ultrasound, Guangzhou, 510080 China; 2 Biomedical Engineering Research Center, Kunming Medical University, Kunming, China; 3 Med-X Research Institute and School of Biomedical Engineering, Shanghai Jiao Tong University, Shanghai, China; 4 Department of Pathology, The First Affiliated Hospital of Sun Yat-Sen University, Guangzhou, China; 5 Biomedical Engineering Department, Peking University, Beijing, 100081 China; 6 Key Laboratory of Carcinogenesis and Translational Research (Ministry of Education/Beijing), Peking University Cancer Hospital & Institute, Beijing, 100142 China

**Keywords:** Ethanol, Hepatocellular carcinoma, Lung neoplasms, Neoplastic cells (circulating), Radiofrequency ablation

## Abstract

**Background:**

We tested the hypothesis that radiofrequency ablation (RFA) for hepatocellular carcinoma (HCC) promotes tumor cell release and explored a method for reducing these effects.

**Methods:**

A green fluorescent protein-transfected orthotopic HCC model was established in 99 nude mice. *In vivo* flow cytometry was used to monitor circulating tumor cell (CTC) dynamics. Pulmonary fluorescence imaging and pathology were performed to investigate lung metastases. First, the kinetics of CTCs during the periablation period and the survival rate of CTCs released during RFA were investigated. Next, mice were allocated to controls, sham ablation, or RFA with/without hepatic vessel blocking (ligation of the portal triads) for evaluating the postablation CTC level, lung metastases, and survival over time. Moreover, the kinetics of CTCs, lung metastases, and mice survival were evaluated for RFA with/without ethanol injection. Pathological changes in tumors and surrounding parenchyma after ethanol injection were noted. Statistical analysis included *t*-test, ANOVA, and Kaplan-Meier survival curves.

**Results:**

CTC counts were 12.3-fold increased during RFA, and 73.7% of RFA-induced CTCs were viable. Pre-RFA hepatic vessel blocking prevented the increase of peripheral CTCs, reduced the number of lung metastases, and prolonged survival (all *p* ≤ 0.05). Similarly, pre-RFA ethanol injection remarkably decreased CTC release during RFA and further decreased lung metastases with extended survival (all *p* ≤ 0.05). Histopathology revealed thrombus formation in blood vessels after ethanol injection, which may clog tumor cell dissemination during RFA.

**Conclusion:**

RFA induces viable tumor cell dissemination, and pre-RFA ethanol injection may provide a prophylactic strategy to reduce this underestimated effect.

**Relevance statement:**

RFA for HCC promotes viable tumor cell release during ablation, while ethanol injection can prevent RFA induced tumor cell release.

**Key points:**

• RFA induced the release of viable tumor cells during the ablation procedure in an animal model.

• Hepatic vessel blocking can suppress tumor cells dissemination during RFA.

• Ethanol injection can prevent RFA-induced tumor cell release, presumably because of the formation of thrombosis.

**Graphical Abstract:**

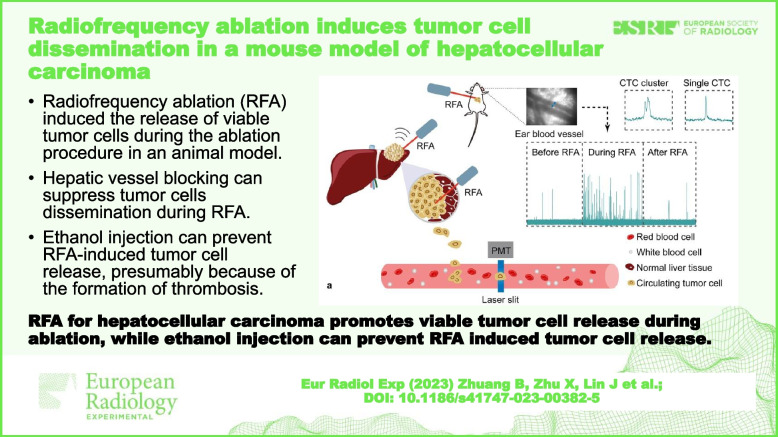

**Supplementary Information:**

The online version contains supplementary material available at 10.1186/s41747-023-00382-5.

## Background

Worldwide, hepatocellular carcinoma (HCC) ranks as the sixth most common form of cancer and the fourth leading cause of cancer-related deaths [1]. In addition to traditional surgical treatments, radiofrequency ablation (RFA) is now commonly used to treat a localized HCC with incorporation into standardized clinical practice algorithms [2, 3]. However, approximately 50 to 70% of HCC patients experience recurrence within 5 years after ablation [4, 5]. Moreover, several reports have raised concerns regarding the unexplained recurrence of aggressive tumors after RFA for HCC [6–8]. However, the exact mechanism underlying postablation recurrence, especially aggressive recurrence, remains unclear.

For many years, numerous studies have demonstrated that some medical manipulation, especially surgical operation, could promote the dissemination of tumor cells into the circulation [6, 7]. Therefore, some surgical techniques, such as no-touch isolation [8], prior feeding, or effluent vessel ligation before en bloc tumor removal [9, 10], have been reported to reduce intraoperative shedding of tumor cells. However, there is still no direct evidence regarding whether RFA can enhance the penetration of cancer cells from the primary tumor into the circulatory system. Thus, it is necessary to investigate the kinetics of ablation-induced tumor cell dissemination and design adjuvant therapeutic strategies to eliminate this effect.

Circulating tumor cells (CTCs), which are shed by cancerous lesions into the bloodstream, are biomarkers of tumor dissemination [11]. Real-time monitoring of CTCs while performing RFA is expected to help answering whether RFA induces tumor cell dissemination. To monitor CTCs, conventional methods usually draw small amounts of blood to isolate and count tumor cells. However, these small volumes of blood allow only a snapshot of CTCs at a specific time point, and those techniques do not allow long-term and dynamical monitoring to explore dynamic changes of CTCs. *In vivo* flow cytometry (IVFC), which can continuously detect fluorescent tumor cells in fast-flowing blood through a laser slit across the artery, is noninvasive and allows real-time assessment of CTCs in the circulatory system of live animals [12, 13]. Previous studies have verified that IVFC presented a high specificity and sensitivity for CTC monitoring [14–16]. Therefore, this technique provides an ideal tool for investigating the dynamic changes of CTCs during RFA.

The aim of this study is to monitor the dynamic changes of CTCs using IVFC and investigated the potential of those tumor cells to form metastasis in a murine orthotopic HCC model. In addition, anhydrous ethanol is commonly used clinically in combination with RFA to increase local efficacy. Given that EI could destruct the vessels within or around the tumors, we attempted to determine whether the combination of EI and RFA could decrease CTC release during RFA.

## Methods

**Fig. 1:**
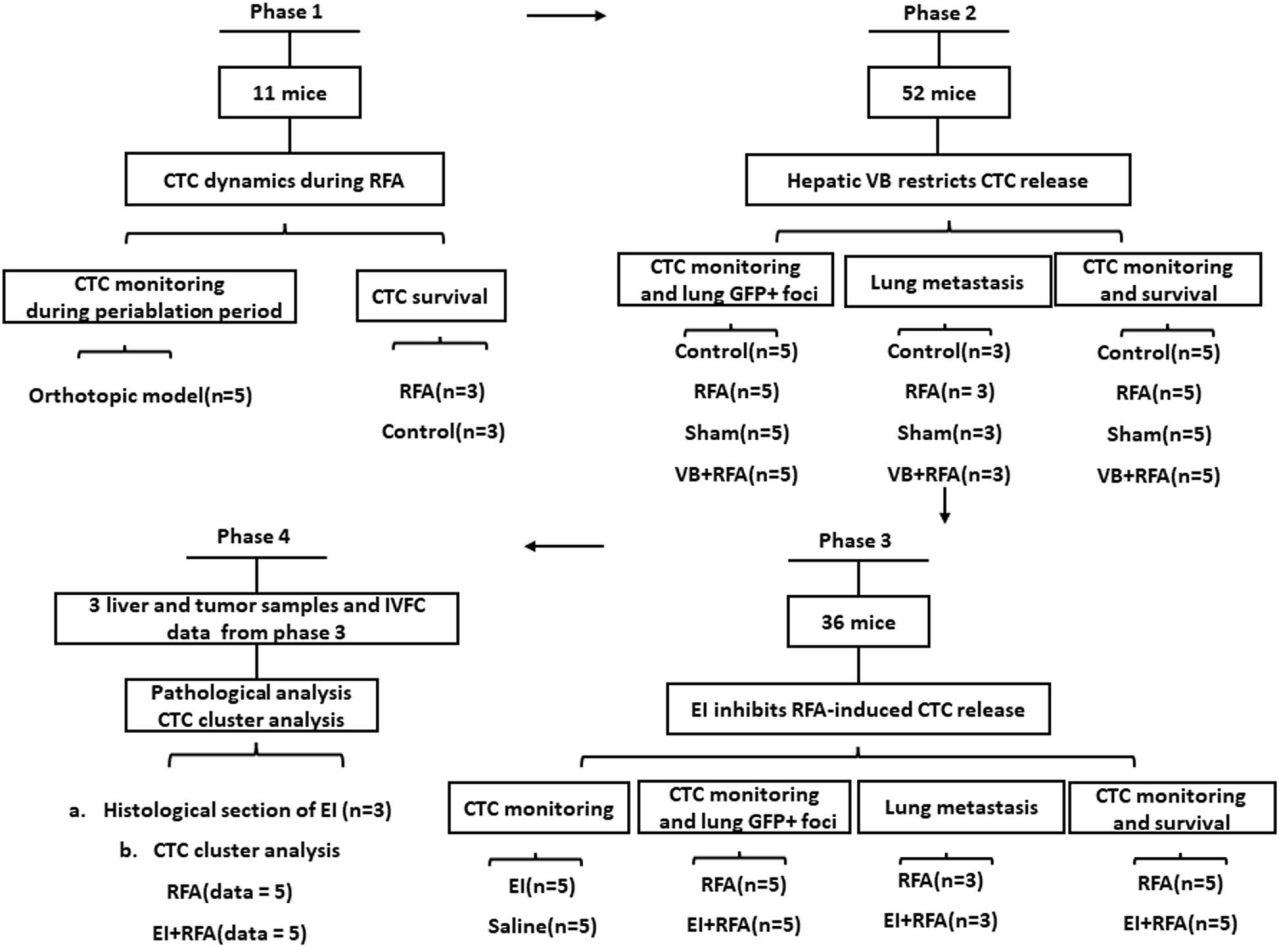
Experimental flowchart

All procedures involving mice were approved by the Ethics Committee of Animal Experiments of Med-X Research Institute and School of Biomedical Engineering at Shanghai Jiao Tong University, China. Our study was performed in four stages in a total of 99 mice (Fig. 1).

### Phase 1: periablational CTC count

We investigated CTC dynamics during the periablation period. First, we continuously monitored CTCs by using IVFC before, during, and after RFA in an orthotopic HCC model. Subsequently, CTCs were monitored until 6 h after RFA (*n* = 5). Second, we extracted blood from the mice in either RFA or control group (*n* = 3 each; total *n* = 6 mice) and performed an apoptosis assay to determine the survival rate of green fluorescent protein (GFP)-positive CTCs.

### Phase 2: the origin and tumorigenic potential of periablational CTC

We assessed whether the increased CTCs arose from the RFA procedure and determined their ability to form successful metastasis. First, mice with orthotopic tumors were randomized into four groups (*n* = 5 each; total *n* = 20 mice): (a) the control group (no treatment), (b) the RFA group, (c) the sham group (laparotomy and electrode placement without energy application), and (d) the VB + RFA group (portal triad and hepatic vein were ligated before RFA). CTC dynamics in each group during the periablation period were monitored by IVFC. The mice were sacrificed after treatment, and lungs were harvested to investigate GFP+ tumor cells using lung imaging. Second, we evaluated CTC dynamics and lung metastatic burden over time after treatment in the four groups mentioned above. CTCs in each group were monitored at 0, 3, 5, 7, 9, 11, and 13 weeks, and overall survival (from the time of treatment to death) was also analyzed in the four groups (*n* = 5 in each group; total *n* = 20 mice). Lungs were harvested 3 weeks after RFA, and pathological sections were performed to investigate lung metastatic burden (*n* = 3 in each group; total *n* = 12 mice).

### Phase 3: EI for decreasing periablational CTC release

We further investigated the influence of EI on the CTC dynamics during RFA. First, we investigated the effects of ethanol injection on CTC release. Ethanol or a corresponding volume of saline was injected into the tumor (*n* = 5 each; total *n* = 10 mice), and CTCs were monitored before and after injection. Second, we evaluated CTC dynamics during the periablation period and GFP+ tumor cells in the lung after ablation in the RFA and EI+RFA groups (*n* = 5 each; total *n* = 10 mice). Third, CTC dynamics *(n* = 5 in each group; total *n* = 10 mice) and lung metastatic burden (*n* = 3 in each group; total *n* = 6 mice) over time after treatment were assessed in the two groups as described in phase 2.

### Phase 4: pathological changes after EI

Three tumor and adjacent liver parenchymal samples from mice in the EI group in the third phase were acquired to analyze pathological changes. IVFC data from mice with > 3 CTCs before RFA in phase 3 were used to analyze the proportion of CTC clusters before, during, and after RFA.

### Cell lines, animal models, and ablation procedure

BALB/c nude mice weighing 20–22 g (6–8 weeks old) were used to establish orthotopic tumor models with GFP-labeled HCCLM3 cells. Once tumors reached target mean diameter of 1 cm, they were randomly allocated to treatment arms. Monopolar RFA was applied by using an S-1500 radiofrequency generator (MedSphere, Shanghai, China). Full details of orthotopic model establish and ablation procedure are described in the supplementary materials.

### IVFC

**Fig. 2:**
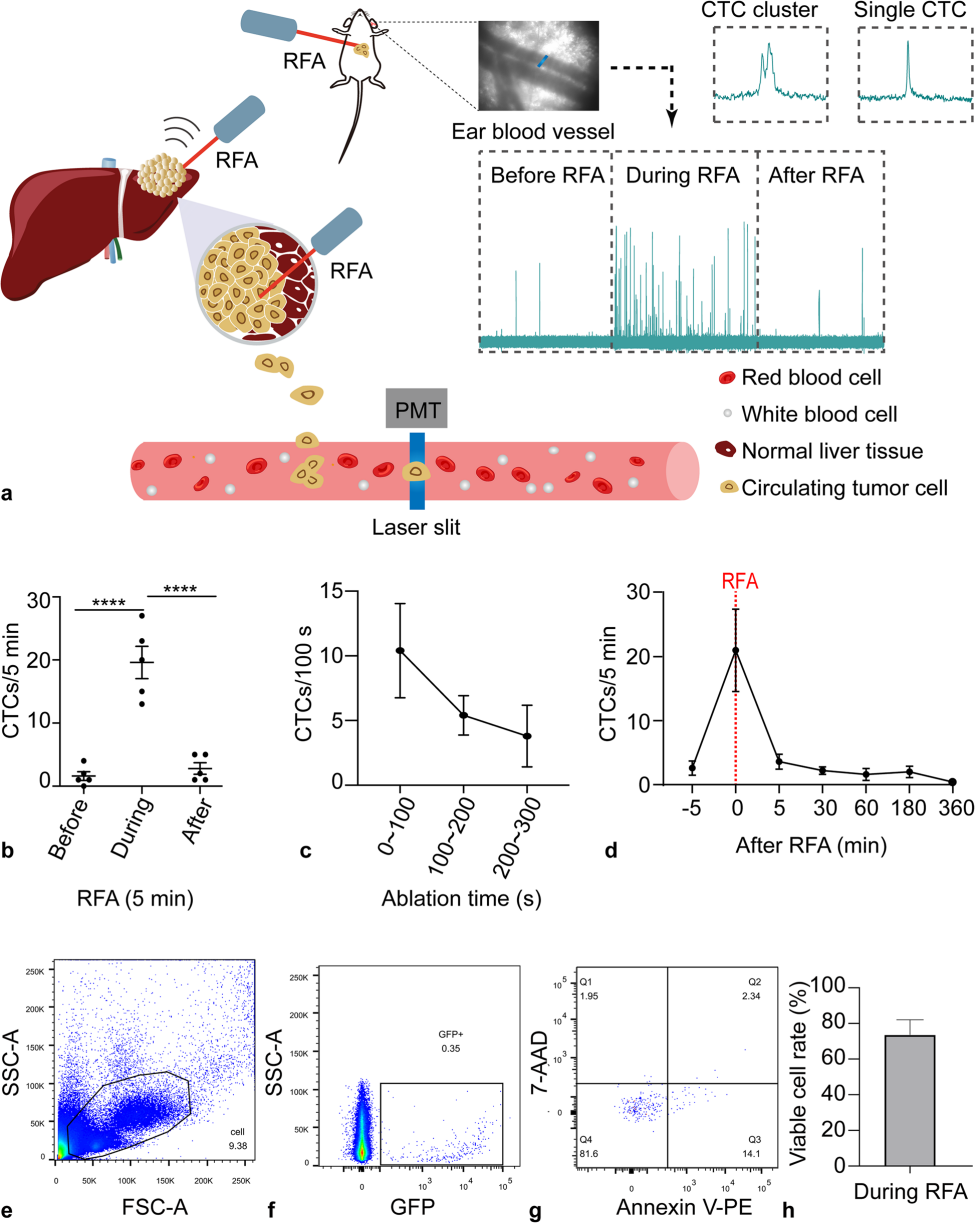
Viable CTCs increase during RFA. **a** Sketch illustrating how RFA provokes tumor cells to release into the circulation. **b** Dynamic changes in CTC counts before, during, and after RFA in orthotopic HCCLM3 tumor-bearing nude mice. **c** Dynamic changes in CTC counts in the orthotopic tumor model during the ablation period. **d** Persistent observation of CTCs after RFA. The red dashed vertical line indicates the time of RFA. Error bars represent the standard error of the means (SEMs) (*n* = 5 for each group. **** *p* < 0.0001. One-way ANOVA followed by Tukey’s *post hoc* test. **e**–**g** Fluorescence-activated cell sorting (FACS) plots indicate the gating strategy and apoptosis analysis for GFP-positive CTCs. **h** Viable cell rate of GFP-positive CTCs induced by RFA *(n* = 3). CTC, Circulating tumor cell; GFP Green fluorescent protein; RFA, Radiofrequency ablation; VB, Vessel blocking

The technical details of IVFC were reported in our previous publication [14] and described in the supplementary materials. For periablation monitoring, IVFC measurement was carried out for 5 min for each mouse. For postablation monitoring, IVFC was performed for 30 min at each time point. Single CTCs and CTC clusters were also identified by their signal patterns (Fig. 2a) [17].

### Annexin V/7-AAD apoptosis assay for confocal and flow cytometry

Blood samples were drawn by cardiac puncture during RFA. The apoptosis rate was determined after sorting the GFP-positive CTCs via a flow cytometer (FACSCalibur, BD) and analyzed with FlowJo software (FlowJo, LLC, USA) (supplementary materials).

### High-resolution lung imaging

The whole lung was removed and placed in a confocal dish. A confocal microscope (Olympus FV1200, Tokyo, Japan) was used to scan the bilateral lung (from the left to the right lung) to randomly acquire five images in unilateral lung (total 10 images for each mouse) for visualizing GFP+ metastatic foci as previously reported [18]. To investigate the lung metastatic foci after treatment, GFP+ fluorescent dots in each view were counted and compared among the groups.

### Histopathology

Mouse tissues were fixed in 4% formalin, embedded in paraffin, and cut into 5-μm sections. All slices were counterstained with hematoxylin-eosin. Antibody against CD31 (Abcam, Cambridge, MA, USA) was used to specifically label vascular endothelial cells. The number and areas of the lung metastasis were calculated in each view using ImageJ software and compared among the different groups.

### Statistical analysis

Statistical analysis was performed with Prism 8.0 software (GraphPad Software). Data are represented as the mean ± standard error of the mean unless differently specified. For the analysis of GFP-positive tumor cells, the fluorescence intensity of five images from each mouse was averaged. An unpaired *t*-test was used to determine significant differences between two groups for data in line with normal distribution and homogeneity of variance. One-way ANOVA followed by Tukey’s *post hoc* test was used to determine the statistical significance of the CTC count in the same group before, during, and after RFA. Survival curves were constructed by the Kaplan-Meier method and compared by the log-rank test. Statistical significance is indicated as a *p*-value < 0.05.

## Results

### Viable CTCs transiently increased during the ablation procedure

IVFC showed that the CTC count was significantly increased during RFA (before *versus* during, 1.6 ± 0.7 *versus* 19.6 ± 2.6, *p* < 0.001) (Fig. 2b). CTC levels during ablation were elevated by 12.3-fold compared to those before RFA. Specifically, the CTC count increased as soon as ablation started, and the increase was observed mainly in the first 200 s of the ablation period (Fig. 2c). Thereafter, the CTC count decreased rapidly to the preablation level after ablation (before *versus* after, 1.6 ± 0.7 *versus* 2.8 ± 0.9, *p* = 0.323) and further decreased to an undetectable level 360 min after ablation (Fig. 2d).

Moreover, we observed both live and apoptotic CTCs in the blood drawn from specimens during RFA (Fig. S 1). Notably, live clustered CTCs were also observed (Fig. S 1). Flow cytometric analysis revealed that 73.7% ± 14.5% of the CTCs induced by RFA were viable (Fig. 2e –h), while CTCs were barely detected in mice that did not undergo RFA (Fig. S 2).

### Hepatic vessel blocking restricts CTC release during ablation

**Fig. 3:**
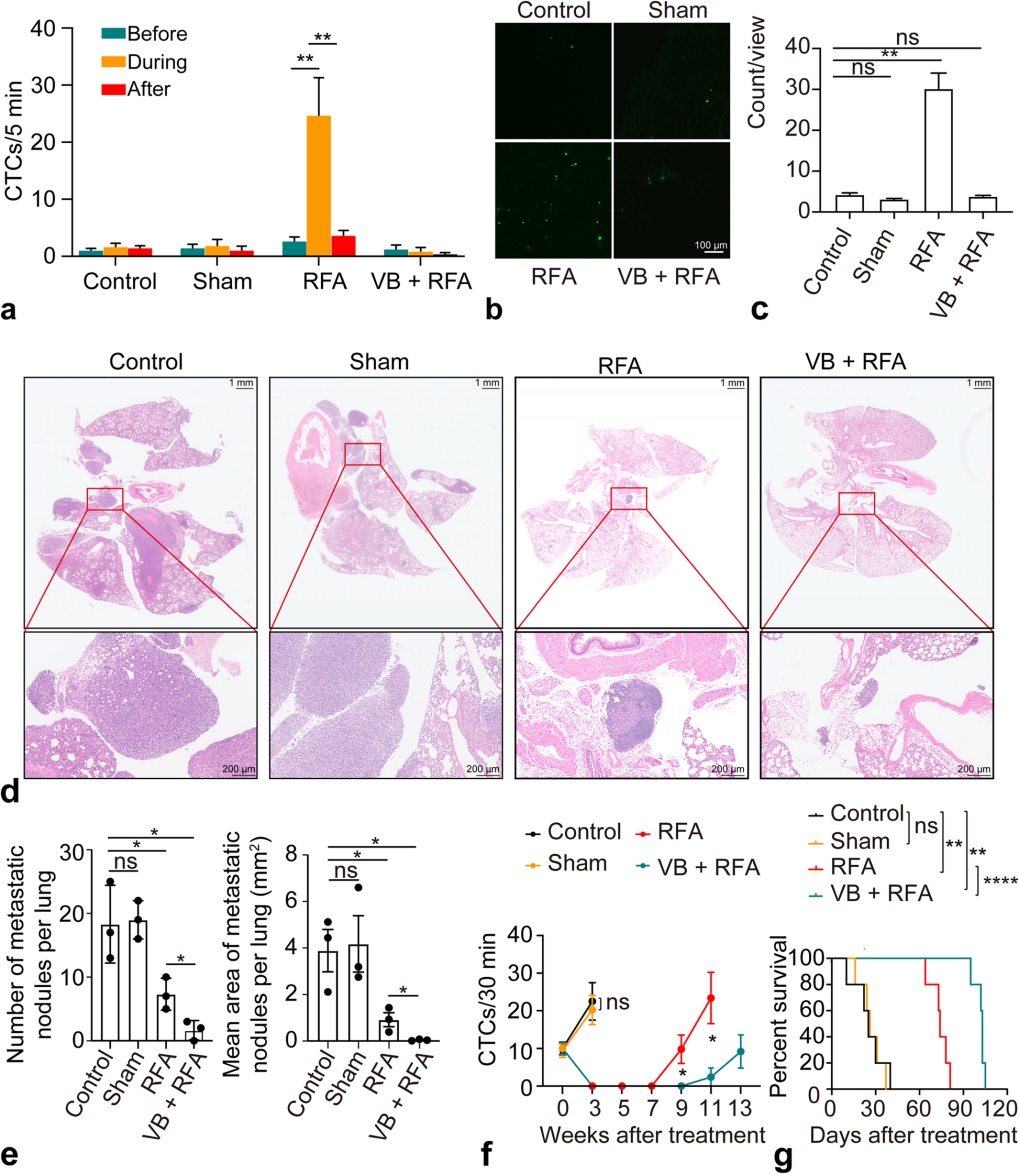
Hepatic vessel blocking prevented CTC release during RFA. **a** Dynamic changes in CTC counts before, during, and after RFA in control, sham ablation, RFA, and vessel blocking (VB) + RFA group *(n* = 5 each group). **b** Representative fluorescence images of mouse lungs from each group after RFA. Scale bar, 100 μm. **c** Lung imaging revealed a higher number of GFP-positive foci in the RFA group after treatment. **d** Representative pathological images of lungs at the indicated timepoints in the control, sham, RFA, and VB + RFA groups. **e** The number and area of the of the lung metastatic foci in the RFA group was significantly higher than that in the VB + RFA group at 5 weeks after RFA *(n* = 3 each group). **f** The CTC count in the RFA group was markedly higher than that in the VB + RFA group at 9 and 11 weeks. **g** Kaplan-Meier survival curves of mice in the four groups. The mice in the VB + RFA group survived longer than that in the RFA group (*n* = 5 each group). *p < 0.05 but ≥ 0.01, **p < 0.01 but ≥ 0.001, ***p < 0.001. CTC, Circulating tumor cell; GFP, Green fluorescent protein; RFA, Radiofrequency ablation; VB, Vessel blocking

The CTC count during the sham procedure (2.0 ± 0.9) was not significantly different from that before (1.6 ± 0.5) or after (1.2 ± 0.6) the sham procedure (*p* = 0.410, *p* = 0.137) (Fig. 3a). Similarly, no significant increase was observed during ablation in the VB + RFA group (before: 1.4 ± 0.6, after: 0.4 ± 0.2 *versus* during :1.0 ± 0.5, *p* = 0.285, *p* = 0.426) (Fig. 3a). The number of GFP+ foci in the lungs was significantly increased 30 min following RFA (RFA *versus* control, 30.1 ± 3.9 *versus* 4.2 ± 0.5, *p* = 0.002); however, this phenomenon was not observed in the sham group (sham *versus* control, 3.1 ± 0.2 *versus* 4.2 ± 0.5, *p* = 0.108) or in the VB + RFA group (VB + RFA *versus* control, 3.8 ± 0.3 *versus* 4.2 ± 0.5, *p* = 0.491) (Fig. 3b, c).

We next investigated the effects of RFA-induced CTCs on metastasis and survival. The initial CTC count before treatment was the same for all groups. No difference in CTC count was observed over time between the sham group and control group (*all p* ≥ 0.05 at each time point) (Fig. 3f), and all mice died within 5 weeks after treatment. The CTC count decreased significantly to near zero in both the RFA and VB + RFA groups after treatment. However, the CTC count in the RFA group started to increase 7 weeks after ablation and was higher than that in the VB + RFA group at 9 weeks (9.8 ± 3.8 *versus* 0.0 ± 0.0, *p* = 0.031) and 11 weeks (23.4 ± 6.8 *versus* 2.4 ± 2.4, *p* = 0.020) after RFA (Fig. 3f). Likewise, the number and area of lung metastatic foci were higher in the RFA group than in the VB + RFA group at 5 weeks (number: 7.3 ± 1.5 *versus* 1.7 ± 0.9, *p* = 0.029; area: 0.92 ± 0.30 *versus* 0.04 ± 0.0.2, *p* = 0.044) after treatment (Fig. 3d, e). Furthermore, the VB + RFA group (median, 103 days) exhibited better survival than the group treated with RFA alone (median, 74 days, log-rank test, *p* < 0.001) (Fig. 3g).

### Ethanol injection inhibited the RFA-induced CTC release

**Fig. 4:**
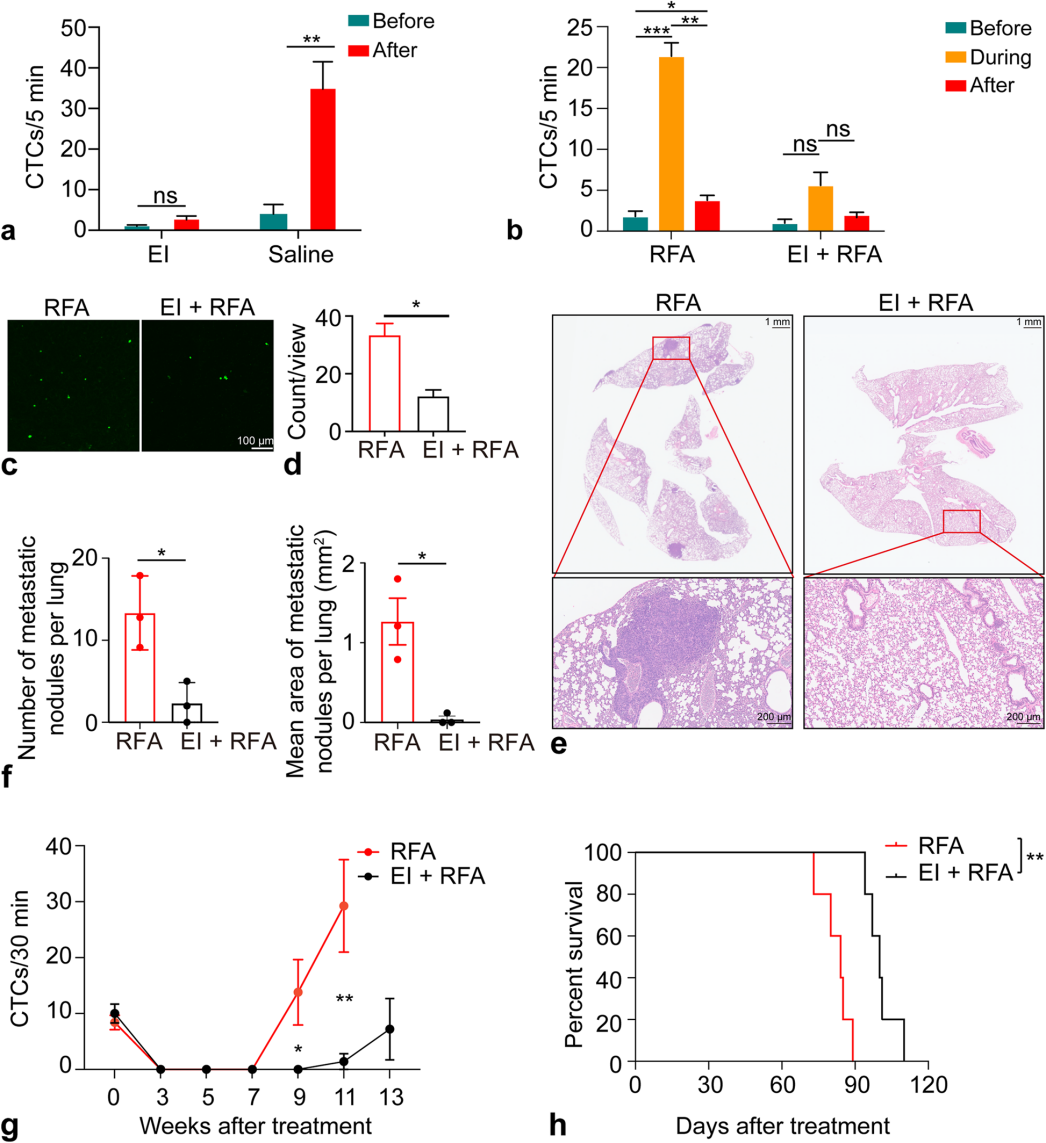
EI decreases CTC release during RFA. **a** Saline injection significantly increased CTCs. However, no significant increase in CTCs was observed in the EI group. **b** There was no significant increase in CTCs during RFA in the EI + RFA group. **c** Representative fluorescence images of lungs after RFA. Scale bar, 100 μm. **d** The lung imaging revealed fewer lung GFP-positive foci in the EI + RFA group than in the RFA group immediately after RFA. **e** Representative pathological images of lungs at the indicated timepoints in the RFA and EI + RFA groups after treatment. Scale bar, 100 μm. **f** The number and area of the lung metastatic foci in the RFA group were significantly higher than that in the EI + RFA group at 7 weeks after RFA. **g** The CTC count in the EI + RFA group was significantly lower than that in the RFA group at 9 and 11 weeks. **h** Kaplan-Meier survival plot of mice showed prolonged survival in the EI+RFA group. *p < 0.05 but ≥ 0.01, **p < 0.01 but ≥ 0.001, ***p < 0.001. CTC, Circulating tumor cell; EI, Ethanol injection; GFP, Green fluorescent protein; RFA, Radiofrequency ablation

The CTC count was significantly increased after saline injection (before *versus* after 4.0 ± 2.3 *versus* 34.8 ± 6.7, *p* < 0.001). However, no significant increase in CTCs was observed after EI (before *versus* after 1.0 ± 0.3 *versus* 2.6 ± 0.9, *p* = 0.141) (Fig. 4a). There was no significant difference in the CTC count during ablation compared with that before or after RFA in the EI + RFA group (before: 1.0 ± 0.5, after: 2.0 ± 0.3 *versus* during: 5.6 ± 1.6, *p* = 0.056, *p* = 0.057) (Fig. 4b). The number of GFP+ foci in the lungs in the EI + RFA group was significantly lower than that in the RFA group at 30 min after ablation (12.1 ± 2.3 *versus* 33.3 ± 4.2, *p* = 0.011) (Fig. 4c, d).

Pathology revealed that the number and area of lung metastatic foci in the EI + RFA group were significantly less than that in the RFA group at 7 weeks (number:13.3 ± 2.6 *versus* 2.3 ± 1.5, *p* = 0.021; area:1.27 ± 0.29 *versus* 0.04 ± 0.04, *p* = 0.014) after treatment (Fig. 4e and f). The CTC count in the RFA group first increased 7 weeks after ablation and was significantly higher than that in the EI + RFA group at 9 weeks (0.0 ± 0.0 *versus* 13.8 ± 5.8, *p* = 0.046) and 11 weeks (1.4 ± 1.4 *versus* 29.3 ± 8.3, *p* = 0.007) after treatment (Fig. 4g). Moreover, the median survival of mice that underwent EI + RFA was greater than that of mice in the RFA group (100 days *versus* 84 days, log-rank test, *p* = 0.002) (Fig. 4h).

### EI prevented tumor cell dissemination through thrombus formation

**Fig. 5:**
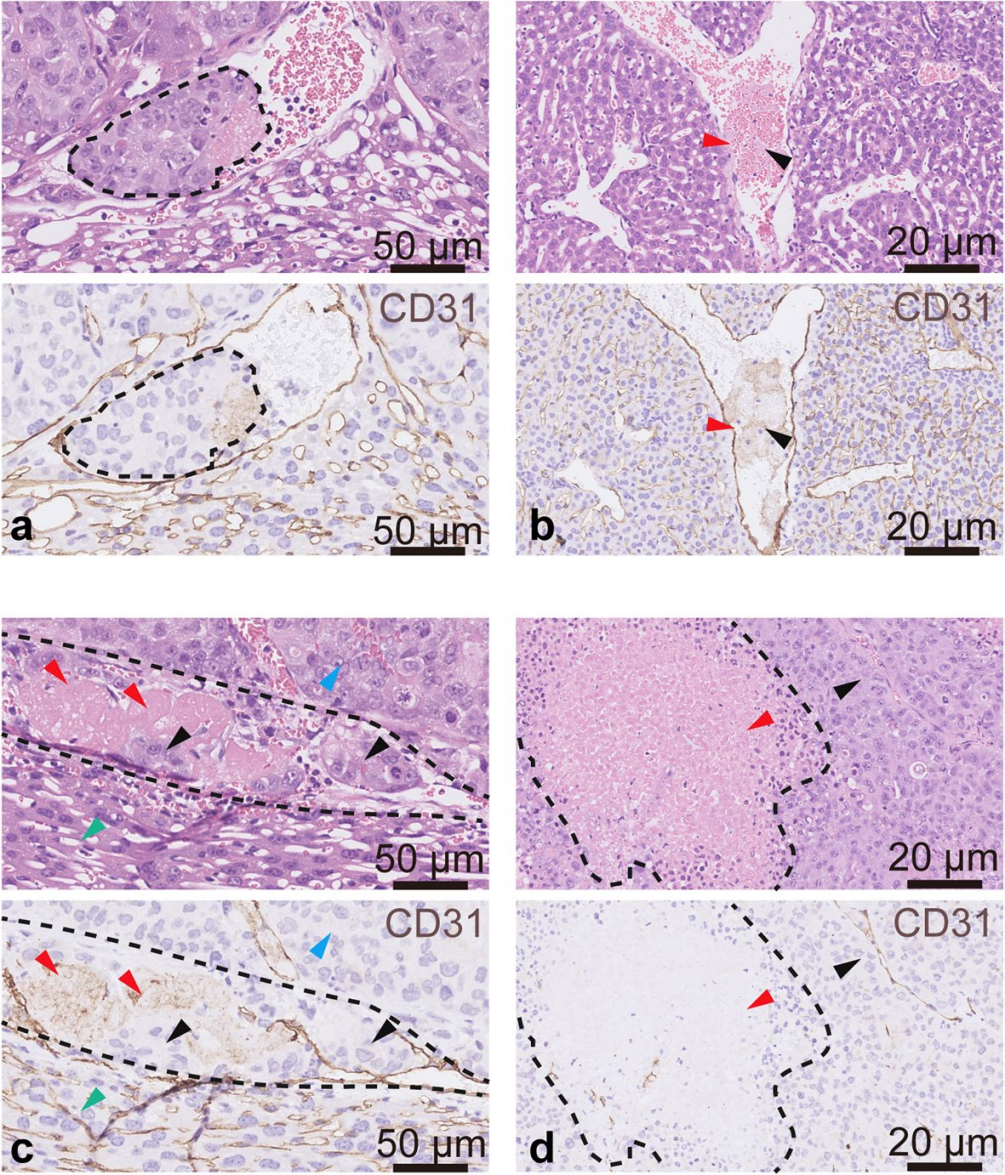
Histopathological findings of the tumor-liver interface in mice with or without EI. **a**–**d** Representative histological images of tumor necrosis and the tumor-liver interface; upper panel: hematoxylin-eosin staining, lower panel: CD31 (blood vessels, brown) staining. **a** A large tumor embolus in the blood vessel. **b** Scattered focal necrosis was observed in live tumor tissues after EI. The red arrow indicates necrotic tumor tissues, and the necrotic tumor cells became granulated, with marked karyolysis. The black arrow indicates surviving tumor tissues. **c** The red arrow indicates a thrombus adhering to the blood vessel wall after EI; the black arrow indicates platelets, cellulose, and reticulocytes in the thrombus. **d** Histological images of the tumor-liver interface after EI. Red arrows indicate thrombi. Black arrows indicate tumor emboli. The green arrow indicates normal liver tissues. The blue arrow indicates tumor tissues. EI, Ethanol injection

We observed abundant tumor embolus in the tumor vessel and the vessels at the tumor-liver interface in the untreated tumor model (Fig. 5a). After EI, multiple areas of focal necrosis were observed in tumor tissue (Fig. 5b). Blood coagulation and partial thrombosis were observed in the interlobular veins and central veins of normal liver tissue (Fig. 5c). Notably, we found that the thrombi clogged the path that the tumor cells used to disseminate in mice that underwent EI (Fig. 5d).

### EI reduced the release of CTC clusters during RFA

**Fig. 6:**
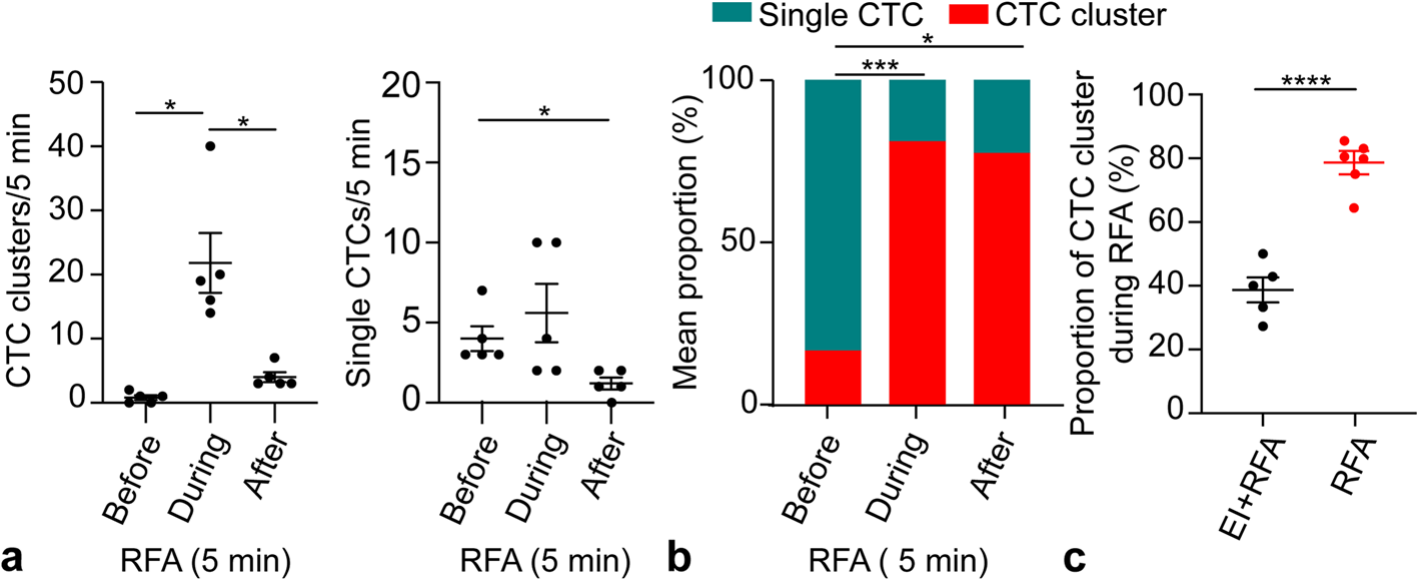
The dynamic changes of CTC clusters during RFA. The count (**a**) and proportion (**b**) of CTC clusters were significantly increased during RFA. **c** The proportion of CTC clusters in the EI + RFA group was significantly lower than that in the RFA group. *p < 0.005 but ≥ 0.001, ***p < 0.001 but ≥ 0.0001, ****p < 0.0001. CTC, Circulating tumor cell; EI, Ethanol injection; RFA, Radiofrequency ablation

CTC clusters (before *versus* during, 0.8 ± 0.4 *versus* 21.8 ± 4.8, *p* < 0.001) but not single CTCs (before *versus* during, 4.0 ± 0.8 *versus* 5.6 ± 1.8, *p* = 0.445) were significantly increased during the RFA procedure (Fig. 6a). The proportion of CTC clusters among total CTC events during RFA was greater than that before RFA (before *versus* during, 16.7% ± 7.0% *versus* 81.1% ± 4.0%, *p* < 0.001) (Fig. 6b). However, the mean proportion of CTC clusters released during RFA in the EI + RFA group was significantly lower than that in the RFA group (78.8% ± 3.7% *versus* 38.7% ± 3.9%, *p* < 0.001) (Fig. 6c).

## Discussion

Usually, ablation is applied to the middle of the tumor and extended around the lesion, which contrasts with the classic surgical principle of “no-touch” tumor resection with a safe margin of surrounding nonneoplastic tissue. This has led to oncologists’ concerns regarding tumor cell spread into the circulation induced by tumor ablation [19]. In accordance with this assumption, several clinical studies have described an increased incidence of diffuse intrahepatic recurrences after RFA of HCC [20–22]. However, direct experimental evidence to support this hypothesis remains limited. Here, we quantitatively evaluated the periablation kinetics of CTCs by using IVFC and attempted to find a clinically useful solution to minimize this effect.

In our study, we observed a temporal but significant hematogenous release of CTCs (12.3-fold increase) during ablation in an orthotopic HCC mouse model. This finding is consistent with a few previous clinical studies demonstrating that tumor cells can be disseminated by RFA [23, 24]. Nonetheless, these previous studies used only small blood samples, which do not well reflect the kinetics of CTCs associated with the intervention methods. Thus, the analysis of the dynamic changes in the CTC levels, as performed in the current study, seems more straightforward and convincing. Currently, the underlying mechanisms that enable tumor cells to be released during RFA are still not well understood. Mechanical stress has long been suspected to enhance the release of viable tumor cells into the circulation during many medical processes [25]. Previous studies have indicated that RFA increases tissue pressure due to the high temperature around the electrode [26, 27], which may promote tumor cells penetration into the blood vessels. Therefore, reducing the intratumor pressure increase during RFA may be beneficial in the clinic to restrict the RFA-induced CTC dissemination.

Tumor cells are believed to circulate in the bloodstream for only a limited period of time with a half-life of only 1 to 2.4 h [28, 29]. Our *in vivo* data show that CTCs increased immediately after RFA began and were cleared within minutes or hours after ablation. Thus, the appropriate window of observation of RFA-induced CTCs was within several hours after ablation, especially during the ablation period. Actually, the precise fate of these CTCs remains unknown. Most of CTCs shed from primary tumor under normal circumstances and die in circulation due to mechanical forces or immune system attack [29]. However, in our studies, 73.7% of CTCs detected during RFA were viable, indicating a large potential to form metastases. Indeed, when we blocked CTC release during ablation by vessel ligation, we found metastases decreased and survival improved. These findings suggested that RFA-induced CTC dissemination resulted in new metastasis, but not just the progression of preexisting lesions.

Notably, we found that both the number and proportion of CTC clusters were significantly elevated during RFA. Histological analysis also revealed large tumor thrombi embedded in tumor vessels before ablation (Fig. 5a). This indicated that RFA was prone to promote the release of clustered tumor cells rather than single tumor cells. CTC clusters are large and are likely to be captured by the capillary bed of the lung or other organs, leading to noticeably fast depletion kinetics. However, compared with single CTC, CTC clusters confer a survival advantage in the vasculature and show enhanced metastatic potential [30]. Therefore, intervention strategies of targeting CTC clusters are necessary for the prevention of RFA-induced metastases.

We further found that RFA-induced CTC dissemination can be successfully inhibited by VB, which has provided a theoretically effective method to eliminate this adverse effect. VB before en bloc tumor removal is a popular surgical technique to prevent the spread of tumor cells into the bloodstream in colorectal [31] or lung [32] cancer. However, VB is difficult to achieve before RFA for HCC because RFA is usually percutaneously performed. EI is performed by directly injecting absolute ethyl alcohol into the tumor, resulting in tumor cell dehydration and coagulation necrosis (Fig. 5b). Moreover, EI has been shown to cause complete vascular occlusion through endothelial damage, vascular thrombosis, erythrocyte sludging, and perivascular necrosis (Fig. 5c, d) [33]. Our study showed that EI before RFA significantly inhibited tumor cell release and subsequently decreased lung metastasis. Tumor cells, especially tumor emboli, may become stuck due to the thrombi and are thus unable to flow into the circulation. Our study suggested EI may also enhance the therapeutic effects of RFA by decreasing tumor cell dissemination during ablation. However, the spread of injected ethanol is largely affected by the capsule or septa of the lesion, which may affect the efficacy. Further confirmatory clinical studies are required to evaluate the effectiveness of the combination of EI and RFA to reduce distant metastasis in clinical practice.

The present study provides a new perspective on postablational recurrence for HCC. Further studies are needed to characterize the full scenario of conditions more extensively, including the following: (a) more tumor types, including those of primary hepatic cancers and other malignancies that metastasize to the liver, such as colorectal cancers, to better understand the generalizability of these findings; (b) different dosimetry and sources of energy (*e.g.*, microwave, cryoablation, laser) performed to determine the extent of variation among different ablation procedures; and (c) alternative procedures to reduce blood supply, such as transhepatic arterial embolization. Notably, achieving transhepatic artery embolization in nude mice poses significant challenges currently. Moreover, this method can only embolize the hepatic artery, allowing for the potential entry of tumor cells into the bloodstream through the hepatic vein during the ablation process.

Of course, our results obtained in a mouse model need verification in humans. However, our findings open support initiation of further studies aiming at preventing RFA-induced CTC release.

In summary, we present experimental evidence that RFA transitorily enhances CTC dissemination during the ablation procedure, which may then lead to an increased metastatic potential. EI before RFA offered a way to reduce this phenomenon and, thus, may be a clinically relevant method for directly addressing this issue.

### Supplementary Information

**Additional file 1:** **Supporting Appendix 1.** Figure Appendix 1: Infrared imaging system was applied to dynamically record the thermal change during RFA in an HCC-cell–derived orthotopic mouse model. **Supporting Appendix 2.** Liver lobe ligation leads to vessel blocking: Contrast-enhanced ultrasound (CEUS) was performed to confirm the blood supply of tumor before and after liver lobe ligation. CEUS were acquired with a Aplio900 US system (Canon Medical Systems) equipped with a 10.0–14.0 MHz linear transducer. A low mechanical index, ranging from 0.07 to 0.09, was used for real-time imaging for CEUS. The contrast agent was Sono-Vue (Bracco, Milan, Italy), a suspension of stabilized sulfur hexafluoride microbubbles in saline. About 100 μL contrast agent was injected via the tail vein. After the tumor exposed, CEUS was performed and showed a microbubble signal enhancement in the tumor. Then, the liver lobe where the tumor was located was ligatured. CEUS showed no microbubble signal enhancement in the tumor, which indicated no blood perfusion in the tumor. Thus, the feeding vessels (both portal triad and hepatic vein) were all blocked. Figure Appendix 2: CEUS finding before and after vessel blocking. a. A tumor of ~1 cm was located in the left lobe of the liver. b. CEUS showed a microbubble signal enhancement in the tumor. c. The liver lobe where the tumor was located was ligatured with a 2-0 silk suture. d. CEUS showed no microbubble signal enhancement in the tumor, which indicated no blood perfusion in the tumor. **Supplementary results 1. **Figure S1: Annexin V/7-AAD double staining assay of peripheral blood extracted during RFA. GFP+ cells are CTCs. Viable cells are Annexin V-/7-AAD-. Cells in early stages of apoptosis are Annexin V+/7-AAD-. Cells in the late stage of apoptosis are Annexin V+/7-AAD+. A. Representative images of CTC, CTC cluster and apoptotic CTC stained with Annexin V/7-AAD. Scale bars: 25μm. **Supplementary results 2. **Figure S2: Gating strategy GFP+ CTCs in peripheral blood extracted from mice in the control group.

## Data Availability

The datasets used and/or analyzed during the current study are available from the corresponding author on reasonable request.

## Abbreviations

CTC: Circulating tumor cell
EI: Ethanol injection
GFP: Green fluorescent protein
HCC: Hepatocellular carcinoma
IVFC: *In vivo* flow cytometry
RFA: Radiofrequency ablation
VB: Vessel blocking
