# Supplementary material for: Radiofrequency ablation induces tumor cell dissemination in a mouse model of hepatocellular carcinoma
Source: Eur Radiol Exp. 2023 Nov 29;7:74. doi: 10.1186/s41747-023-00382-5 (PMC10686970; doi:10.1186/s41747-023-00382-5)
Supplement: Supplementary file 1 — Additional file 1: Supporting Appendix 1. Figure Appendix 1: Infrared imaging system was applied to dynamically record the thermal change during RFA in an HCC-cell–derived orthotopic mouse model. Supporting Appendix 2. Liver lobe ligation leads to vessel blocking: Contrast-enhanced ultrasound (CEUS) was performed to confirm the blood supply of tumor before and after liver lobe ligation. CEUS were acquired with a Aplio900 US system (Canon Medical Systems) equipped with a 10.0–14.0 MHz linear transducer. A low mechanical index, ranging from 0.07 to 0.09, was used for real-time imaging for CEUS. The contrast agent was Sono-Vue (Bracco, Milan, Italy), a suspension of stabilized sulfur hexafluoride microbubbles in saline. About 100 μL contrast agent was injected via the tail vein. After the tumor exposed, CEUS was performed and showed a microbubble signal enhancement in the tumor. Then, the liver lobe where the tumor was located was ligatured. CEUS showed no microbubble signal enhancement in the tumor, which indicated no blood perfusion in the tumor. Thus, the feeding vessels (both portal triad and hepatic vein) were all blocked. Figure Appendix 2: CEUS finding before and after vessel blocking. a. A tumor of ~1 cm was located in the left lobe of the liver. b. CEUS showed a microbubble signal enhancement in the tumor. c. The liver lobe where the tumor was located was ligatured with a 2-0 silk suture. d. CEUS showed no microbubble signal enhancement in the tumor, which indicated no blood perfusion in the tumor. Supplementary results 1. Figure S1: Annexin V/7-AAD double staining assay of peripheral blood extracted during RFA. GFP+ cells are CTCs. Viable cells are Annexin V-/7-AAD-. Cells in early stages of apoptosis are Annexin V+/7-AAD-. Cells in the late stage of apoptosis are Annexin V+/7-AAD+. A. Representative images of CTC, CTC cluster and apoptotic CTC stained with Annexin V/7-AAD. Scale bars: 25μm. Supplementary results 2. Figure S2: Gating strategy GFP+ CTCs in per [file 41747_2023_382_MOESM1_ESM.docx]

**Radiofrequency Ablation Induces Tumor Cell Dissemination in a Mouse Model of Hepatocellular Carcinoma**

**ELECTRONIC SUPPLEMENTARY MATERIAL**

**Cell lines, animal models and ablation procedure**

GFP-labeled HCCLM3 cells (obtained from the Liver Cancer Institute, Zhongshan Hospital, Fudan University) were grown in Dulbecco’s modified Eagle’s medium (DMEM) (Gibco, Carlsbad, CA, USA) supplemented with 10% fetal bovine serum (Gibco) at 37°C and 5% CO2. BALB/c nude mice (purchased from Shanghai Slac Laboratory Animal Co., Ltd.) weighing 20–22 g (6-8 weeks old) were used to establish tumor models. For the orthotopic tumor model, a small tumor cube with a volume of approximately 1 mm^3^ was implanted into the left lateral lobe of the liver as previously reported[1]. For all mice, in vivo ultrasound imaging (VisualSonics Vero 7700TM, Toronto, Canada) was performed every 1 to 2 days to observe tumor sizes. Once tumors reached the target mean diameter of 1 cm, they were randomly allocated to treatment arms.

Monopolar RFA was applied by using an S-1500 radiofrequency generator (MedSphere, Shanghai, China). A thermocouple at the tip of the RF electrode constantly measured the local ablation temperature, thereby enabling proper generator manipulation. Ablation was performed for 4-5 min at 70°C using a 17-gauge single electrode (MedSphere, Shanghai, China) with a 10-mm active tip.

Infrared camera (FLUKE, TIS 20+) was used to monitor and record dynamic thermal change of the tumor, and to ensure central temperature >60°C for necrosis of the target tumors as previously reported[2](Supporting Appendix 1).

Mice were anesthetized with ketamine/xylazine (100 mg/10 mg/kg, Sigma Aldrich, St. Louis, MO, USA). For orthotopic tumor ablation, the tumor in the left liver lobe was exposed to permit direct-contact ablation. Small quantities of saline were applied to wet the exposed tissue during ablation, preventing it from drying and adhering to the abdominal wall. For sham ablation, the RF electrode was inserted into the tumors without power output. In the VB+RFA group, the liver lobe where the tumor was located was ligatured before ablation with a 2-0 silk suture. Thus, the feeding vessels (both portal triad and hepatic vein) were all blocked(Supporting Appendix 2). In the EI + RFA group, approximately 0.2 ml of dehydrated ethanol was slowly injected, first into the bottom of the tumor and then into the rest of the tumor. Injections were performed slowly over 30 seconds to ensure uniform diffusion of the ethanol. The amount of ethanol injected was determined according to the size of the tumor as previously described[3]. After injection, no leakage of ethanol around the needle or out of the tumor was observed. At least one minute after EI, RFA was performed as mentioned above.

**IVFC**

Briefly, an artery of the mouse ear was imaged with a charge coupled device (CCD). A 488-nm laser was shaped into a slit and imaged across the ear artery. When fluorescently labeled cells flowed through the laser slit, fluorescence was excited and detected with a photomultiplier tube (PMT). The hemodynamics of the CTCs were measured by positioning the anesthetized mice on the stage to detect GFP+ CTCs. A fluorescence signal with multiple peaks and a peak-to-peak value greater than the bandwidth of noise in a time window of 40 ms was generated by a CTC cluster event, while a fluorescence signal with a single peak was generated by a single CTC

**Annexin V/7-AAD apoptosis assay for confocal and flow cytometry**

The samples were lysed with red blood cell lysis buffer (Beyotime Biotechnology) for 3 min and centrifuged for 5 min at 400 g. The pellet was washed once with cold PBS and a second time with Annexin V binding buffer (BD Biosciences, CA, USA). Then, the cells were resuspended in Annexin V binding buffer containing 5 μL of APC-Annexin V (for confocal microscopy) or PE-Annexin V (for flow cytometry) and 5 μL of 7-AAD (BD Biosciences) for 10 min at room temperature in the dark.

**Supporting Appendix 1**


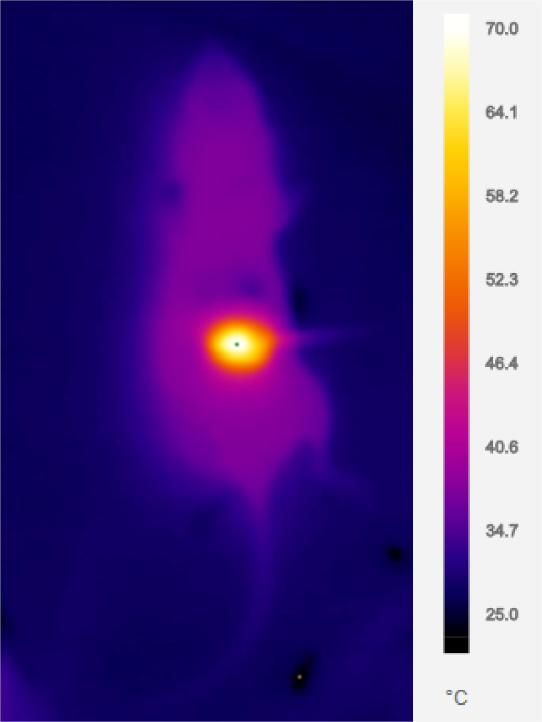


**Figure Appendix 1:** Infrared imaging system was applied to dynamically record the thermal change during RFA in an HCC-cell–derived orthotopic mouse model

**Supporting** **Appendix 2. Liver lobe ligation leads to vessel blocking**

Contrast-enhanced ultrasound (CEUS) was performed to confirm the blood supply of tumor before and after liver lobe ligation. CEUS were acquired with a Aplio900 US system (Canon Medical Systems) equipped with a 10.0–14.0 MHz linear transducer. A low mechanical index, ranging from 0.07 to 0.09, was used for real-time imaging for CEUS. The contrast agent was Sono-Vue (Bracco, Milan, Italy), a suspension of stabilized sulfur hexafluoride microbubbles in saline. About 100 μL contrast agent was injected via the tail vein. After the tumor exposed, CEUS was performed and showed a microbubble signal enhancement in the tumor. Then, the liver lobe where the tumor was located was ligatured. CEUS showed no microbubble signal enhancement in the tumor, which indicated no blood perfusion in the tumor. Thus, the feeding vessels (both portal triad and hepatic vein) were all blocked.

**
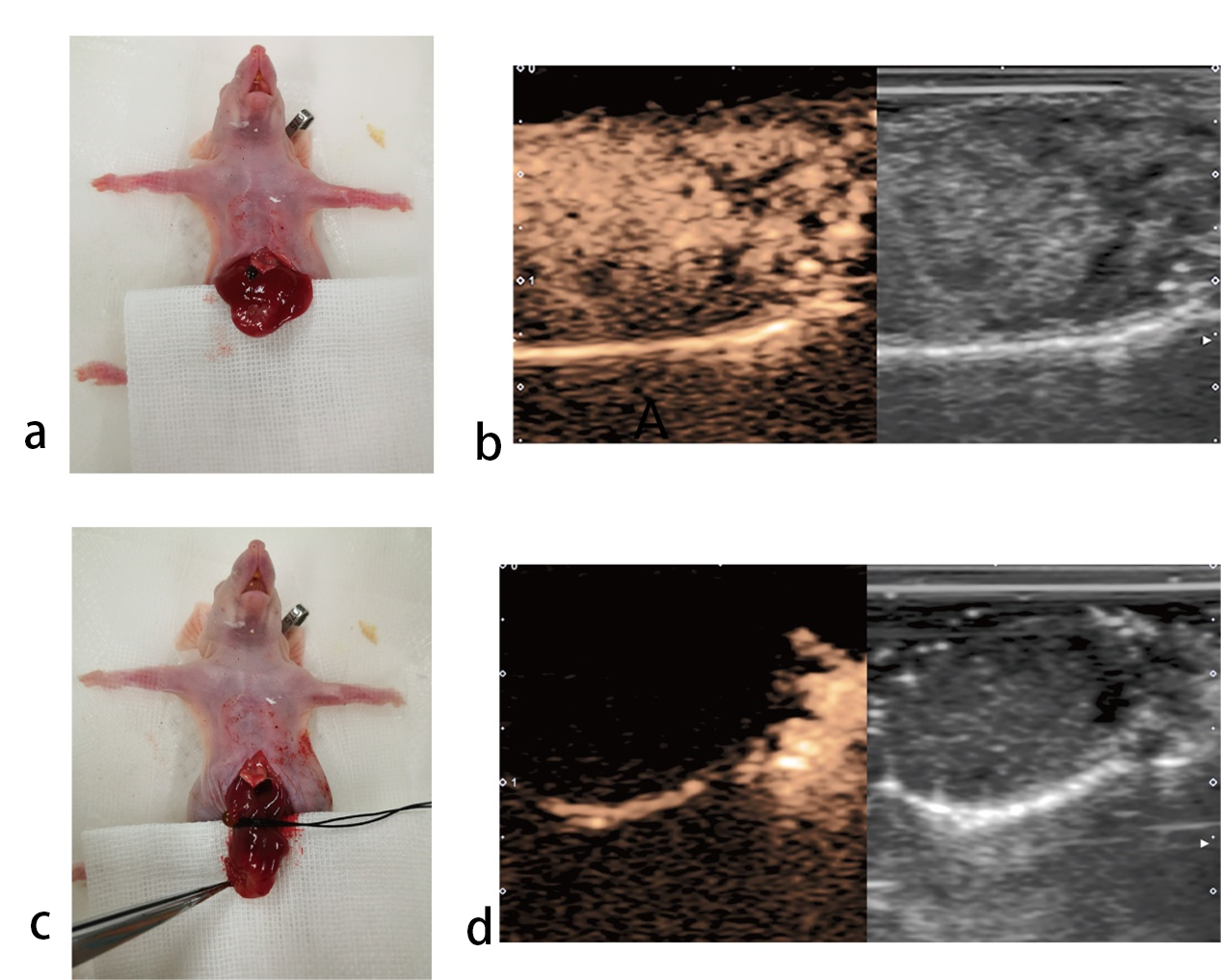
**

**Figure Appendix 2:** CEUS finding before and after vessel blocking. a. A tumor of ~1cm was located in the left lobe of the liver. b. CEUS showed a microbubble signal enhancement in the tumor. c. The liver lobe where the tumor was located was ligatured with a 2-0 silk suture. d. CEUS showed no microbubble signal enhancement in the tumor, which indicated no blood perfusion in the tumor.

**Supplementary results 1**


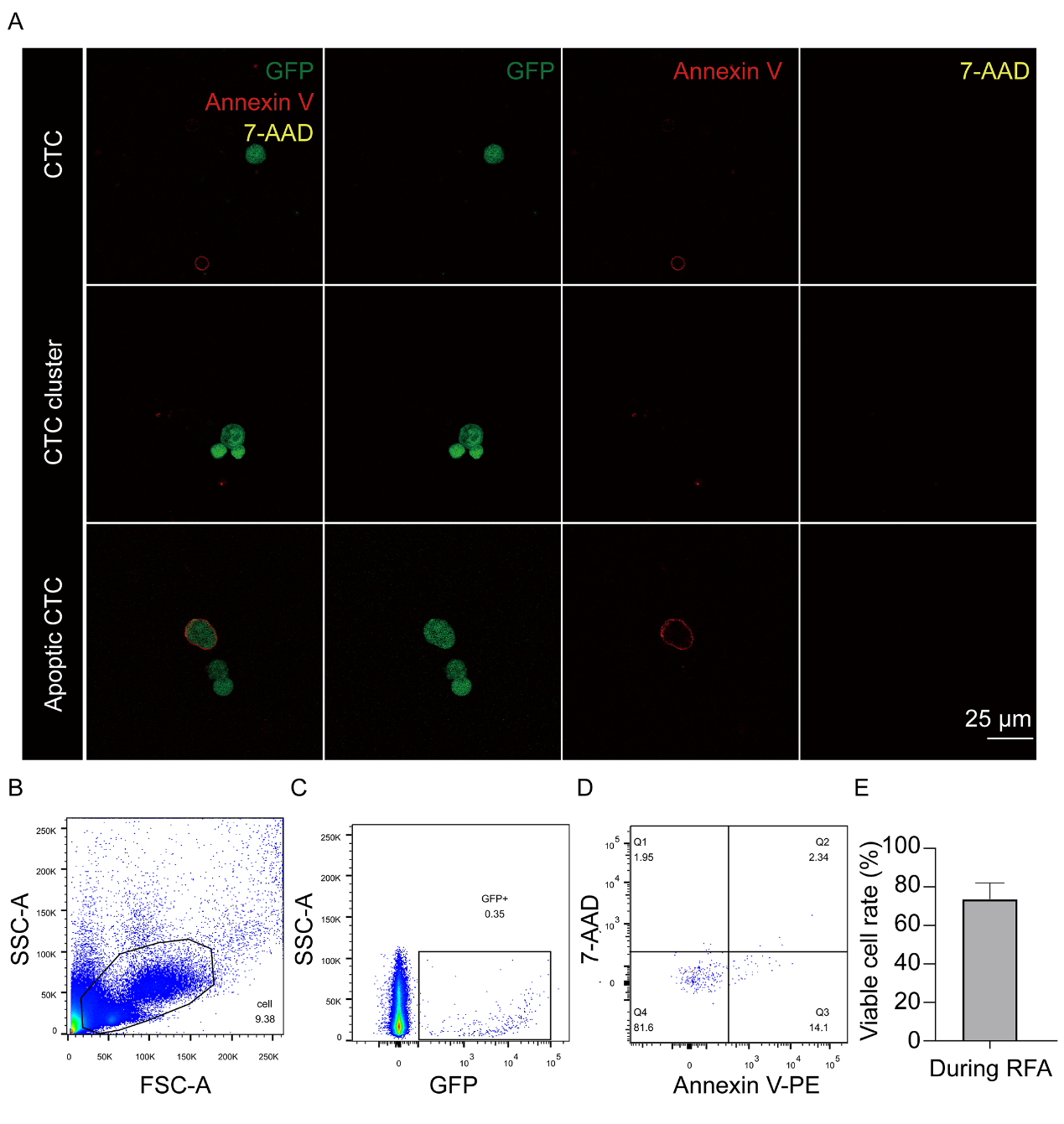


**Figure S1: Annexin V/7-AAD double staining assay of peripheral blood extracted during RFA.** GFP+ cells are CTCs. Viable cells are Annexin V-/7-AAD-. Cells in early stages of apoptosis are Annexin V+/7-AAD-. Cells in the late stage of apoptosis are Annexin V+/7-AAD+. A. Representative images of CTC, CTC cluster and apoptotic CTC stained with Annexin V/7-AAD. Scale bars: 25μm.

**Supplementary results 2**


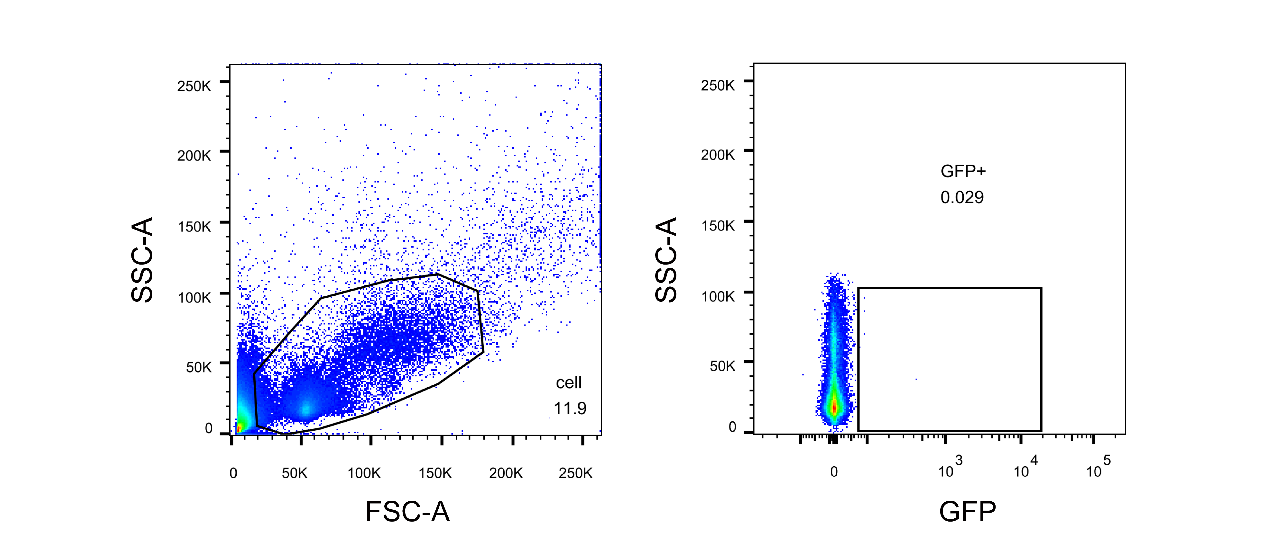
**Figure S2: Gating strategy GFP+ CTCs in peripheral blood extracted from mice in the control group.**

1 Fan ZC, Yan J, Liu GD et al (2012) Real-time monitoring of rare circulating hepatocellular carcinoma cells in an orthotopic model by in vivo flow cytometry assesses resection on metastasis. Cancer Res 72:2683-2691

2 Su T, Huang M, Liao J et al (2021) Insufficient Radiofrequency Ablation Promotes Hepatocellular Carcinoma Metastasis through m(6) A mRNA Methylation Dependent Mechanism. Hepatology 74:1339-1356

3 Huang G, Lin M, Xie X et al (2014) Combined radiofrequency ablation and ethanol injection with a multipronged needle for the treatment of medium and large hepatocellular carcinoma. Eur Radiol 24:1565-1571
